# The Association between Baseline Subjective Anxiety Rating and Changes in Cardiac Autonomic Nervous Activity in Response to Tryptophan Depletion in Healthy Volunteers

**DOI:** 10.1097/MD.0000000000003498

**Published:** 2016-05-13

**Authors:** Chih Yin Hsiao, Hsin Chun Tsai, Mei Hung Chi, Kao Chin Chen, Po See Chen, I Hui Lee, Tzung Lieh Yeh, Yen Kuang Yang

**Affiliations:** From the Department of Psychiatry, National Cheng Kung University Hospital (CYH, HCT, MHC, KCC, PSC, IHL, TLY, YKY), College of Medicine; Addiction Research Center (CYH, KCC, PSC, IHL, TLY, YKY), National Cheng Kung University, Tainan; Department of Psychiatry (HCT, KCC, YKY), National Cheng Kung University Hospital, Dou-Liou Branch, Yunlin; and Institute of Behavioral Medicine (YKY), College of Medicine, National Cheng Kung University, Tainan, Taiwan.

## Abstract

The aim of this study was to investigate the influence of serotonin on anxiety and autonomic nervous system (ANS) function; the correlation between subjective anxiety rating and changes of ANS function following tryptophan depletion (TD) in healthy volunteers was examined. Twenty-eight healthy participants, consisting of 15 females and 13 males, with an average age of 33.3 years, were recruited.

Baseline Chinese Symptom Checklist-90-Revised and ANS function measurements were taken. TD was carried out on the testing day, and participants provided blood samples right before and 5 hours after TD. ANS function, somatic symptoms, and Visual Analogue Scales (VASs) were determined after TD. Wilcoxon signed rank test and Spearman ρ correlation were adapted for analyses of the results.

The TD procedure reduced total and free plasma tryptophan effectively. After TD, the sympathetic nervous activity increased and parasympathetic nervous activity decreased. Baseline anxiety ratings positively correlated with post-TD changes in sympathetic nervous activity, VAS ratings, and physical symptoms. However, a negative correlation with post-TD changes in parasympathetic nervous activity was found.

The change in ANS function after TD was associated with the severity of anxiety in healthy volunteers. This supports the fact that the effect of anxiety on heart rate variability is related to serotonin vulnerability. Furthermore, it also shows that the subjective anxiety rating has a biological basis related to serotonin.

## INTRODUCTION

Anxiety disorders contribute 3.5% of all years lived with disability,^1^ and have a high comorbidity with depression, hypertension,^2^ and cardiovascular disease.^3^ Previous studies have suggested that impaired regulation of the heart by the autonomic nervous system (ANS) involves pathophysiological links.^4^ ANS dysfunction, which can present as increased sympathetic activity or decreased parasympathetic activity, may also be related to higher peripheral somatosensory arousal and, in turn, to increased experience of somatic symptoms.^5^

The serotonergic neurons of the central nervous system (CNS) regulate mood, appetite, and sleep.^6^ A prominent role for serotonin (5-HT) in anxiety and depression is well acknowledged.^7–8^ Since 5-HT1A knockout mice have been found to exhibit abnormal maturation of cardiorespiratory control,^9^ serotonin might influence ANS regulation of the heart. Studies in animals have suggested that serotonergic projections to the periaqueductal gray region play an important role in suppressing the behavioral and autonomic correlates of panic.^10^

Through a combination of a low-protein diet and a tryptophan-free drink containing large neutral amino acids (LNAAs), the tryptophan depletion (TD) test has been proven to be an effective method of lowering the production of central serotonin.^11–15^ During the test, the mean nadir of tryptophan was observed in the sixth hour after consumption.^16^ Subjects have been found to react differently to TD manipulation depending on their level of serotonin vulnerability.^17^ Previous research has shown that TD challenge leads to the exacerbation of transient symptoms, such as anxiety,^11–14,18^ impulsivity,^19–20^ and impaired decision-making,^21^ in several subgroups of patients, including those with generalized anxiety disorder (GAD), social anxiety disorder, substance abuse, attention deficit hyperactivity disorder (ADHD), and major depressive disorder (MDD).^11–14,18–20,22^

Reduced heart rate variability (HRV) has been proven to be a more useful measure of cardiac autonomic function than other peripheral markers, such as blood pressure or heart rate.^23–24^ Reduced HRV has been observed not only in patients with chronic physical illnesses,^25–26^ but also in patients suffering from anxiety disorders,^27–29^ and in unmedicated MDD patients with comorbid GAD.^30^ Owing to multiple influencing factors, including the effects of medication and disease itself on HRV, there are no consistent data to support that serotonin activity has an effect on ANS function. HRV is not changed after sertraline loading in healthy volunteers.^31–32^ In patients with other diagnoses, such as panic disorder or acute depression, antidepressant treatment did not consistently increase HRV.^33–35^ Therefore, the effects of serotonin activity on anxiety and ANS function warrant further investigation.

Some TD studies have indicated that 5-HT has an effect on ANS function^15,18,22^; however, other studies have reported no effects.^36–37^ Booji et al^18^ applied the Fourier transform cardiac ANS index in remitted depressed patients and found that TD decreased HRV only in those with a history of suicidal ideation. This implies that reduced HRV in depression after TD may be limited to individuals with characteristics such as impulsive or aggressive behaviors.

Most studies on the effect of serotonin on the ANS have been performed on patients with anxiety or depression disorders. Clarification of the relationship between anxiety and serotonin in healthy participants is necessary. This study aimed to examine correlates of the degree of reaction to TD in healthy volunteers. We hypothesized that the degree of change in cardiac ANS function after TD is associated with anxiety.

## MATERIALS AND METHODS

### Participants

Our study was conducted at National Cheng Kung University Hospital between 2010 and 2013 to explore serotonin vulnerability. In the cohort study of healthy people, participants were recruited through an advertisement approved by the ethical committee for human research. The inclusion criteria were (i) age 20 to 65 years; (ii) no past or current psychiatric disorder; (iii) no history of a psychiatric disorder in any first-degree relative; (iv) no major medical condition, such as cancer, heart disease, or organ failure; (v) no prescribed drugs that might interfere with the objectives of the study, such as beta blockers; and (vi) no history of illicit drug or psychoactive medication use, as confirmed by a negative urine screen. A senior psychiatrist conducted the Mini International Neuropsychiatric Interview with all participants.^38^ Totally, 43 participants were recruited in this cohort study. Only participants younger than 45 years (also applied to males for balance) were analyzed in this study to prevent any potential effect of menopause. The final sample consisted of 28 subjects (13 males, 15 females). Among the 15 participants excluded (5 males and 10 females), 11 were older than 45 years and 4 did not have baseline ANS recordings.

### Procedures

Informed consent, demographic data (such as age, sex, and body mass index [BMI), Chinese Symptom Checklist-90-Revised (SCL-90-R) results, and ANS function data were obtained at baseline.

The TD challenge was administered to every participant, and was timed to coincide with the follicular phase in women. Participants adhered to a 24-hour low-protein diet and began fasting at 10:00 pm the night before the TD test. At 9:00 am on the day of testing, participants ingested the TD amino acid mixture. Blood samples for the measurement of total and free tryptophan were taken shortly before and 5 hours after administration of the drink. ANS function, somatic symptoms, and VASs related to TD were measured at 2:00 pm.

The amino acid drink was composed of 15 amino acids without l-tryptophan, as in previous studies.^13,39^ Because of their unpleasant taste, participants took the sulfur-containing amino acids (methionine, threonine, and arginine) in capsule form 15 minutes before consuming the remaining amino acids in drink form. The drink was prepared by mixing amino acid powder with 300 mL of room-temperature water. The amino acid solution was flavored with approximately 5 mL of chocolate-flavored syrup.

### Assessments

#### Measurement of Cardiac Autonomic Function

Cardiac autonomic function was determined using an ANSWatch wrist monitor, which calculated the HRV (Taiwan Scientific Corporation, Taipei, Taiwan). This device employed multiple piezo-electrical sensors in the cuff to directly measure the blood pressure waveforms in the radial artery.

All the ANSWatch tests were performed in a quiet laboratory with soft light and a minimum level of stimulation to prevent any external influence on the participants. All participants were asked to avoid any activity for 60 minutes before the test, and sat quietly in the laboratory for at least 30 minutes before the test. Each ANSWatch test was completed within about 7 minutes. The data were then downloaded to a personal computer and analyzed using ANSWatch software. The accuracy of ANSWatch in terms of the correlation coefficient for HRV parameters was in the range of 0.90 to 1.0, as compared with the use of electrocardiography as the control.^40^

HRV was analyzed through frequency and time domain methods. The intervals between adjacent normal R waves (NN intervals) and the quantified cyclic fluctuations of R-R were measured over the recording period. The parameters were low frequency (LF, 0.04–0.15 Hz), high frequency (HF, 0.15–0.4 Hz), and LF/HF (ratio of LF to HF) in the frequency domain, as well as pNN50 (the proportion of NN50s in the total number of NNs, wherein NN50 is the number of pairs of successive NNs that differ by >50 ms) and the HRV triangular index in the time domain.^23^ LF is an index of sympathetic activity, whereas HF is an index of parasympathetic activity. The LF/HF ratio represents the sympathovagal balance. Higher LH/HF ratios reflect increased sympathetic activity or decreased parasympathetic modulation.^41^ pNN50 approximates to HF in the frequency domain.^23,42–43^ The HRV triangular index resembles the total power as a total ANS activity index.^44^ ANSWatch testing was performed twice in this study, at both baseline and 5 hours post-TD.

#### Plasma Tryptophan Levels

Total and free tryptophan in plasma and the ratio of total tryptophan to LNAAs and tryptophan/LNAA were assessed to evaluate the efficacy of the TD procedure. Collected blood samples were centrifuged for 15 minutes at 4°C and 3000 rpm immediately. Plasma was frozen at –70°C until analysis. Immediately after thawing, 20 μL of 70% perchloric acid was added to 400 μL of plasma, followed by centrifugation for 30 minutes at 20,000*g* at 4°C, causing precipitation of the plasma proteins. To detect free tryptophan, plasma was filtered using an Amicon Ultra-0.5 Centrifuge Filter (10K device).^45^ Liquid chromatography-tandem mass spectrometry (API 2000 LC/MS/MS system) was used following a previously reported method^46^ to analyze the total and free tryptophan and other amino acids.

### Psychological Assessment

#### The Chinese Symptom Checklist-90-Revised

This study used the Symptom Checklist-90-Revised (SCL-90-R, published by the Clinical Assessment division of the Pearson Assessment & Information group), a reliable and validated scale.^47–49^ The checklist assesses the following 9 primary psychological symptoms: somatization, obsessive–compulsion, interpersonal sensitivity, depression, anxiety, hostility, phobic anxiety, paranoid ideation, and psychoticism. Only the 10-item anxiety subscale was utilized in this study, with higher scores representing higher anxiety levels. A previous analysis reported that the SCL-90-R has a 76.5% hit rate in distinguishing anxiety disorder from depressive disorder: patients with anxiety scored more highly in the anxiety subscale.^50^

#### Somatic Symptoms

Our study adopted the Panic Attack Symptom Scale,^51^ which includes a list of somatic symptoms and has been used previously in TD research.^13^ Participants rated 23 symptoms on a 4-point scale (1, not at all; 2, mild; 3, moderate; and 4, severe). To analyze the number of somatic symptoms after TD, we recoded the scale into a 2-point scale (0, not at all; 1, mild to severe). Scale scores were summed, with higher scores representing greater severity of somatic symptoms after TD.

#### VASs for Mood States

We applied VASs that were adapted from previous research^51^ and have been used in other TD studies.^11,14–15^ The participants rated the changes in 13 different mood states after TD: talkative, happy, sad, drowsy, anxious, nervous, energetic, calm, fearful, depressed, angry, high, and mellow. The scales were scored on a 0- (not at all) to 100-mm (most) line, wherein participants placed a perpendicular mark on the left-hand side that corresponded to his or her present state as compared with baseline.

### Statistical Analysis

The data were analyzed using SPSS software 17.0 (SPSS Inc, Chicago, IL). Means and standard deviations (SDs) were calculated for the descriptive analysis of ANS function, both at baseline and after TD. Because the scores of the SCL-90-R anxiety subscale, VASs, somatic symptoms, and parameters of cardiac ANS functions were not of a normal distribution, nonparametric statistical analysis was conducted. The effects of age, sex, and BMI on the distributions of cardiac ANS function indexes in 3 conditions (baseline, after TD, and change rate) were examined by comparing the difference between groups (age: equal or above mean/under mean age [n: 17/11], sex: male/female [n: 13/15], BMI: obesity/nonobesity [n: 6/22], according to the standard of the Ministry of Health and Welfare in Taiwan). The Mann-Whitney *U* test was used in 2 groups’ comparison. The Wilcoxon signed rank test was adapted to compare baseline and post-TD ANS function. Two-tailed Spearman ρ correlation analysis was carried out to examine the associations between baseline anxiety score, VASs, somatic symptoms, and change in ANS function after TD.

## RESULTS

The demographic characteristics of 28 participants are presented in Table 1.

**TABLE 1:**
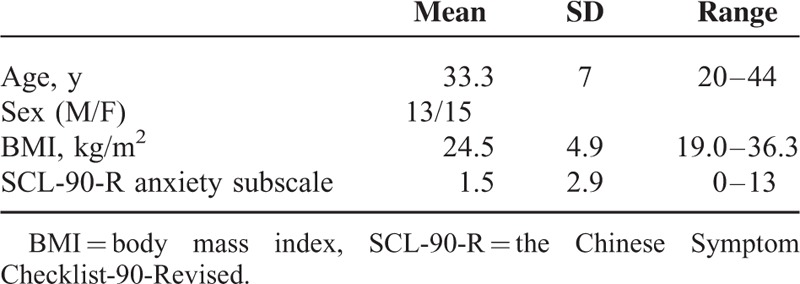
Demographic Data and Baseline Anxiety Rating of 28 Participants

The TD procedure resulted in an 87.2% reduction in both total and free tryptophan, in addition to a 95.3% reduction in the tryptophan/LNAA ratio, which would result in an effective reduction in serotonin. After TD, the mean number of symptoms was 2.75 (range = 0–8; SD = 2.05), and disgust (n = 24, 86%), frequent urination (n = 12, 43%), and poor appetite (n = 10, 37%) were the most common symptoms. According to VAS rating, 7 participants (25%) experienced more anxiousness and 8 of them (28.6%) felt much nervous. Age and BMI did not affect the distributions of HRV indexes in all 3 conditions (*P*s > 0.06) except obesity group with lower HRV triangular index (*P* = 0.04). Although females had higher HF, lower LF, and LF/HF at baseline and after TD compared with males (*P*s < 0.011), no significant sex difference in pNN50 and HRV triangular index was noted (*P*s > 0.37). The change rate of ANS functions in terms of these 5 indexes showed no sex difference (*P*s > 0.46).

The comparison of HRV parameters at baseline and post-TD is shown in Table 2. PNN50, HRV triangular index, and HF all decreased following TD as compared with baseline, whereas LF and LF/HF increased. Indexes representing parasympathetic tone, such as pNN50 and the HF power spectrum, were significantly lowered following TD. Conversely, parameters related to the balance of sympathetic/parasympathetic activity, such as LF/HF, were higher after TD. Four of 5 effect sizes for changes in HRV indexes following TD showed medium effect (Table 2).

**TABLE 2:**
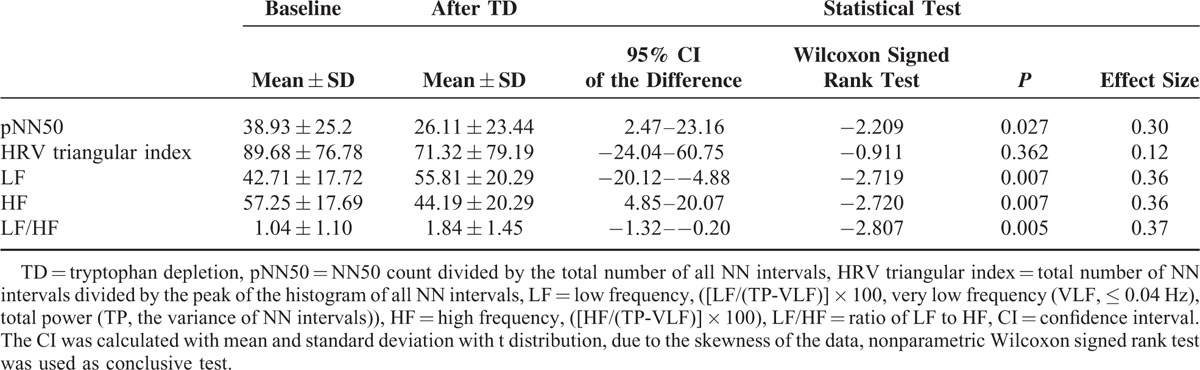
Comparison of Cardiac Autonomic Function at Baseline and After TD

The correlations between the baseline anxiety rating and cardiac ANS parameters, somatic symptoms, and VASs after TD are shown in Table 3. The baseline anxiety score was not significantly associated with any of the baseline cardiac ANS indexes. However, there were significantly negative correlations between the baseline anxiety score and the change rates in the following cardiac ANS parameters: pNN50 and HRV triangular index. In addition, a positive correlation between baseline anxiety score and LF/HF was also noted. Likewise, the baseline anxiety score was positively associated with subjective mood change in terms of the anxiety and nervousness VAS scoring, as well as symptoms after TD. The higher the anxiety score at baseline, the higher the VAS anxiety, the VAS nervousness rating, and the number of physical symptoms after TD.

**TABLE 3:**
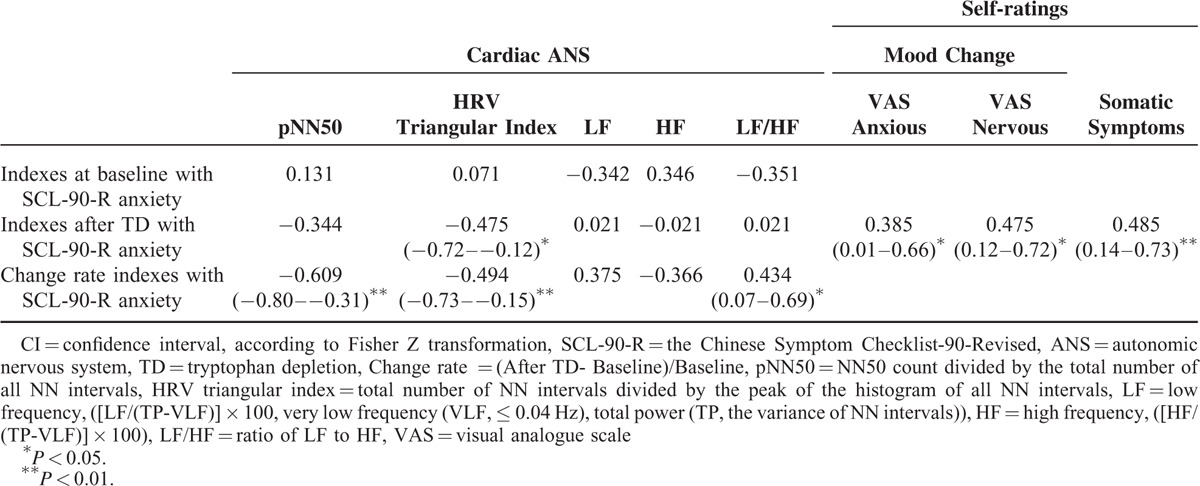
Correlations Between SCL-90-R Anxiety Subscale at Baseline and Cardiac ANS Functions/Self-ratings (95% CI [in Parentheses])

## DISCUSSION

In this study, the TD procedure reduced plasma tryptophan levels effectively, and this is in accordance with previous researches.^13,16,52^ Even all participants had received cardiac ANS function measurements through standardized procedure, as HRV indexes in our study had wide distributions, which also appeared in previous neurophysiology studies.^53^ Factors including age, sex, and BMI might influence the results of ANS functions, and should be considered and managed carefully. Most time- and frequency-domain indexes of HRV except LF/HF ratio decrease with age and these values further decrease until fourth decade of life.^53^ In our study, age did not affect the distribution of HRV significantly. One of the speculated reasons could be we had set a narrow range of age for inclusion to avoid the effects of menopause. The sex difference in the frequency-domain HRV indexes at baseline and following TD is in line with previous findings.^53^ However, there was no sex effect on the change ratio of HRV indexes in response to TD. As regarding to BMI, we only found the correlation with HRV triangular index; BMI was not related to the change ratio of any HRV index responding to TD as sex. Therefore, age, sex, and BMI were not needed to be controlled as confounding factors.

Following TD, the HRV triangular index and the power of HF decreased; however, the power of LF and the LF/HF ratio increased. This phenomenon indicates that the imbalance of sympathovagal tone and the attenuation of parasympathetic activity related to decreased central serotonin concentration are not only found in participants with depression or anxiety, as reported in previous research,^15,18,35,54–55^ but also in healthy participants. Furthermore, the level of the decrease in parasympathetic nervous activity and the imbalance in sympathovagal tone after TD were correlated with the baseline anxiety rating in our healthy volunteers.

In addition to the previously reported reduction in HRV in individuals with anxiety disorders,^27–28^ and in healthy volunteers with extreme trait anxiety,^56^ we found attenuated parasympathetic activity and reduced HRV induced by TD in our healthy volunteers without a personal or family history of psychiatric disorders or any complaints regarding ANS dysfunction. Although most previous research has concentrated on comparing the HRV with a control group, in this study, intraindividual comparisons were applied to measure ANS changes in healthy participants. The method reduced the effect of interference from medication or methodological issues. We speculate that the higher self-rated anxiety score in healthy people might be a marker of serotonin vulnerability.

The blood pressure variability caused by respiration would cause HRV via the arterial baroreflex because the reflex reacts to any blood pressure increase or decrease by a heart rate decrease or increase.^57^ According to Kellett et al,^58–59^ serotonin depletion or 5-HT receptor blocking attenuates baroreflex gain. This mechanism could partly explain the reduced HRV observed following TD. Other mechanisms could also play roles, including reduced production of central serotonin, which influences the modulation of the limbic system through the 5-HT_1A_ receptor,^60^ and a set of neural structures associated with emotional regulation, such as the prefrontal cortex.^61–62^

The participants with higher anxiety scores not only gave higher self-reports of nervousness and physical symptoms after TD, but also had a decreased HRV index. Some studies have reported that the mood-lowering effect of TD may be because of the characteristics of participants,^63^ which implied a core problem of subjective reporting. The concordance of subjective self-reported VASs and symptoms with objective HRV measurements suggests that HRV is a suitable index for measurement of the effects of the TD procedure. Clinical rating scales might not be sensitive enough to detect psychological or somatic responses^11,64^; however, advanced tools such as functional brain imaging cannot be applied widely owing to cost and accessibility.^21^ In line with the findings that a reduced HRV is present in healthy volunteers both with recent stressful life events^65^ and task applications related to anxiety and depression,^66^ changes in HRV parameters have been suggested to be indicators of serotonin vulnerability in studies with a challenge test design.

Previous research has provided inconsistent findings on whether HRV is reduced because of the psychopathology of depression, or whether the reduction is driven by antidepressant medication. In a recent study, Kemp et al^30^ reported that HRV was reduced most in relation to comorbid GAD, not depression severity. Furthermore, previous evidence has suggested that anxiety, not depression, drives the reported HRV reductions in MDD.^67^ It has also been found that a reduced HRV in mice with a high anxiety trait can be reversed by anxiolytic drug treatment.^68^ The aforementioned evidence suggests the importance of investigating the role of anxiety in HRV, and accorded with our results regarding the effect of anxiety on HRV in situations of a decreased central serotonin level. To better understand the complicated pathology of ANS dysfunction in depression and anxiety patients, further research needs to take serotonin vulnerability into account.

Our results must be interpreted with caution because of the following potential limitations. First, the sample sizes were relatively small and the age range of the participants was limited to between 20 and 45 years. We were not able to compare the differences of demographic characteristics between the study subjects and those who declined or refused to join the study. Second, we did not include a placebo as a control for comparison with TD. Third, we applied the symptom checklist only after TD; the ratings were not used both pre- and post-TD. Lastly, the HRV level is sensitive to environmental and personal factors, which may not have been included.

In conclusion, our findings indicate that serotonin vulnerability could be a potential explanation for ANS dysfunction in healthy participants with higher baseline anxiety score. The effect of serotonin vulnerability on anxiety should be validated in further studies.
